# Methotrexate-associated lymphoproliferative disorder in the stomach and duodenum: a case report

**DOI:** 10.1186/s12876-019-0982-4

**Published:** 2019-04-25

**Authors:** Haruka Toyonaga, Masashi Fukushima, Naoto Shimeno, Tetsuro Inokuma

**Affiliations:** Department of Gastroenterology, Kobe City Medical Center General Hospital, 2-1-1 Minatojima-minamimachi, Chuo-ku, Kobe, Hyogo 650-0047 Japan

**Keywords:** Methotrexate-associated lymphoproliferative disorders, Diffuse large B-cell lymphoma, Rheumatoid arthritis

## Abstract

**Background:**

Methotrexate-associated lymphoproliferative disorder (MTX-LPD) can present as a benign lymphoid proliferation or a malignant lymphoma in patients taking MTX. Almost 50% of MTX-LPD cases show spontaneous remission after withdrawal of MTX treatment. Studies have suggested that the hyper-immune state of rheumatoid arthritis, the immunosuppressive state associated with MTX, and the carcinogenicity of the Epstein-Barr virus might contribute to MTX-LPD development. Although most cases of MTX-LPD occur at extranodal sites, few cases of MTX-LPD affecting the stomach and duodenum have been reported. To our knowledge, no other study has reported on the endoscopic observations of dramatic withdrawal and appearance of multiple digestive tract lesions in a short period of time. Herein, we report the clinical course and imaging findings of our case, which may be useful for understanding the pathological condition of MTX-LPD.

**Case presentation:**

We describe the case of a 70-year-old woman with MTX-LPD of the stomach and duodenum. Disease regression was temporarily achieved after cessation of MTX treatment; however, it subsequently recurred, and complete response was only achieved after six cycles of rituximab, cyclophosphamide, hydroxydaunorubicin, oncovin, and prednisolone (R-CHOP) chemotherapy.

**Conclusions:**

The first-choice therapy for patients taking MTX who develop suspected MTX-LPD should be the withdrawal of MTX treatment. Even after remission is achieved, patients should be kept under careful observation, and if the disease recurs, chemotherapy should be commenced promptly.

## Background

Methotrexate-associated lymphoproliferative disorder (MTX-LPD) can present as a benign lymphoid proliferation or a malignant lymphoma in patients taking MTX. In 1991, Ellman and colleagues reported the first case of lymphomas in a patient with rheumatoid arthritis (RA) treated with MTX [1]. The World Health Organization categorizes MTX-LPD under “other iatrogenic immunodeficiency-associated lymphoproliferative disorders.” MTX-LPD consists mainly of diffuse large B-cell lymphoma (DLBCL; 35–60% of cases) and classical Hodgkin’s lymphoma (12–25% of cases) [2]. Approximately 40–50% of MTX-LPD cases occur at extranodal sites, such as the skin, salivary glands, lungs, digestive tract, liver, and spine [3]. Although spontaneous remission of MTX-LPD after MTX withdrawal occurs in approximately 50% of cases [4], chemotherapy may be needed to treat lymphoma recurring or persisting after stopping MTX treatment.

Here, we describe a case of MTX-LPD in the stomach and duodenum that temporarily resolved after the cessation of MTX treatment. In order to achieve complete response after recurrence of disease, prompt chemotherapy was required. To our knowledge, there are no other reports on the endoscopic observations of dramatic withdrawal and the appearance of multiple digestive tract lesions within a short period. The imaging findings along with the patient’s clinical course in this case may prove useful for understanding the pathological condition of MTX-LPD.

## Case presentation

A 70-year-old woman presented to the clinic with a history of epigastric distress. Her medical history was significant for *Helicobacter pylori* infection, which was resolved five years prior; and RA, for which she had been taking MTX (6 mg per week) for the past 6 months. Her symptoms were investigated with esophagogastroduodenoscopy (EGD), which initially revealed no abnormality apart from atrophic gastritis. Following a two-month course of acid-suppressing drugs, she remained symptomatic; therefore, a repeat EGD was conducted, which revealed the emergence of multiple elevated lesions. As a result, she was referred to our hospital.

Physical examination at that time revealed the abdomen to be soft and flat, with no hepatosplenomegaly or lymphadenopathy. Laboratory tests showed elevated levels of lactate dehydrogenase (312 IU/L; reference range, 120–250 IU/L) and soluble interleukin-2 receptor (sIL-2R) (1430 IU/mL, reference range, 145–520 IU/mL). The lymphocyte count was 2375/μl (19%, reference range, 19–61%).

**Fig. 1:**
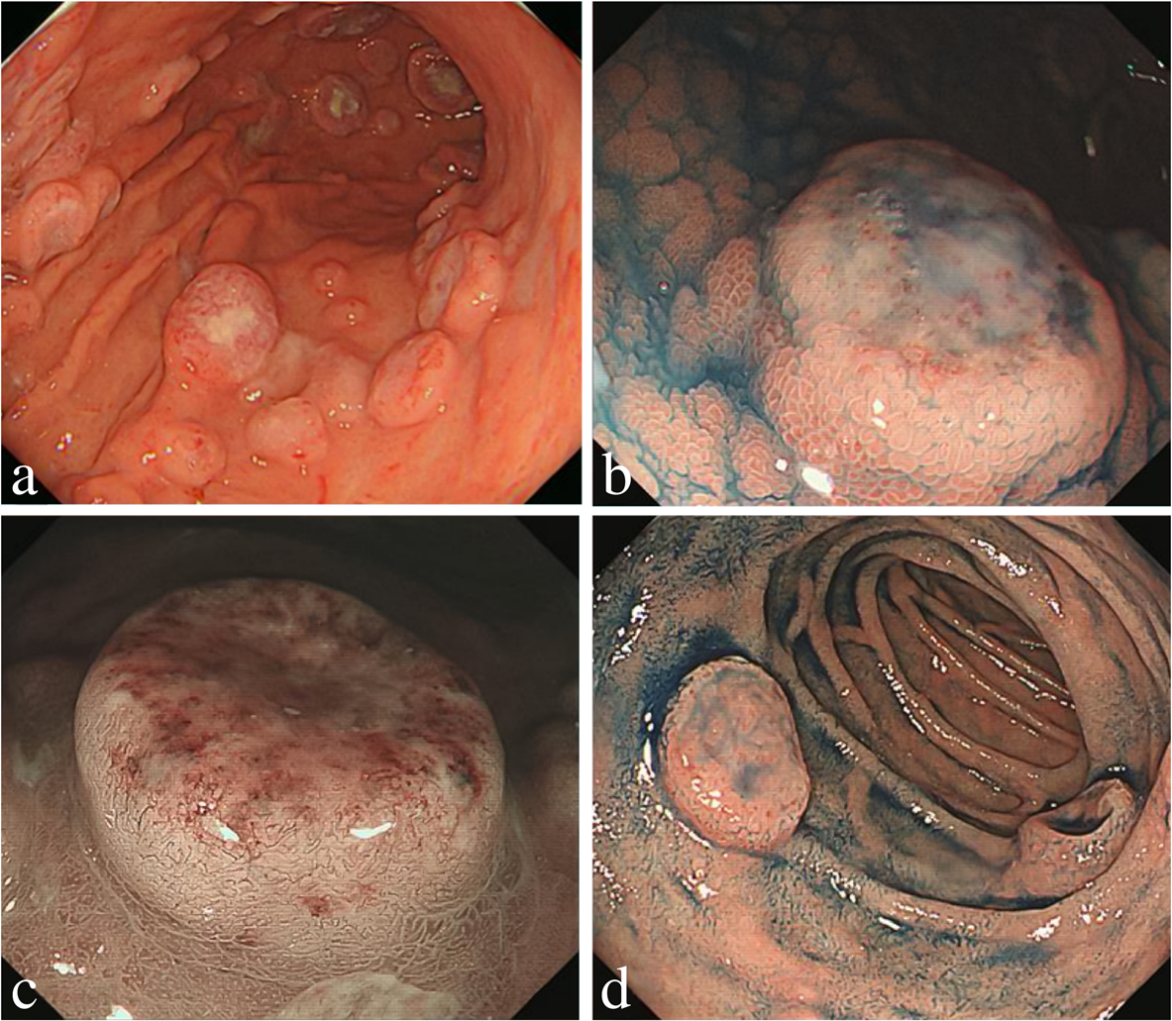
Endoscopic images of the stomach and duodenum affected by MTX-LPD. **a**, Before the withdrawal of MTX treatment, there were multiple dish-like lesions in the stomach. **b**, Indigo carmine spraying revealed that the lesion rise was relatively steep, the surface structure was equivalent to that of the background mucosa, and ulceration with white coat was observed in the central part of the lesion. **c**, Narrow band imaging revealed meandering irregular microvessels without loops. **d**, There were dish-like lesions also in the duodenum

**Fig. 2:**
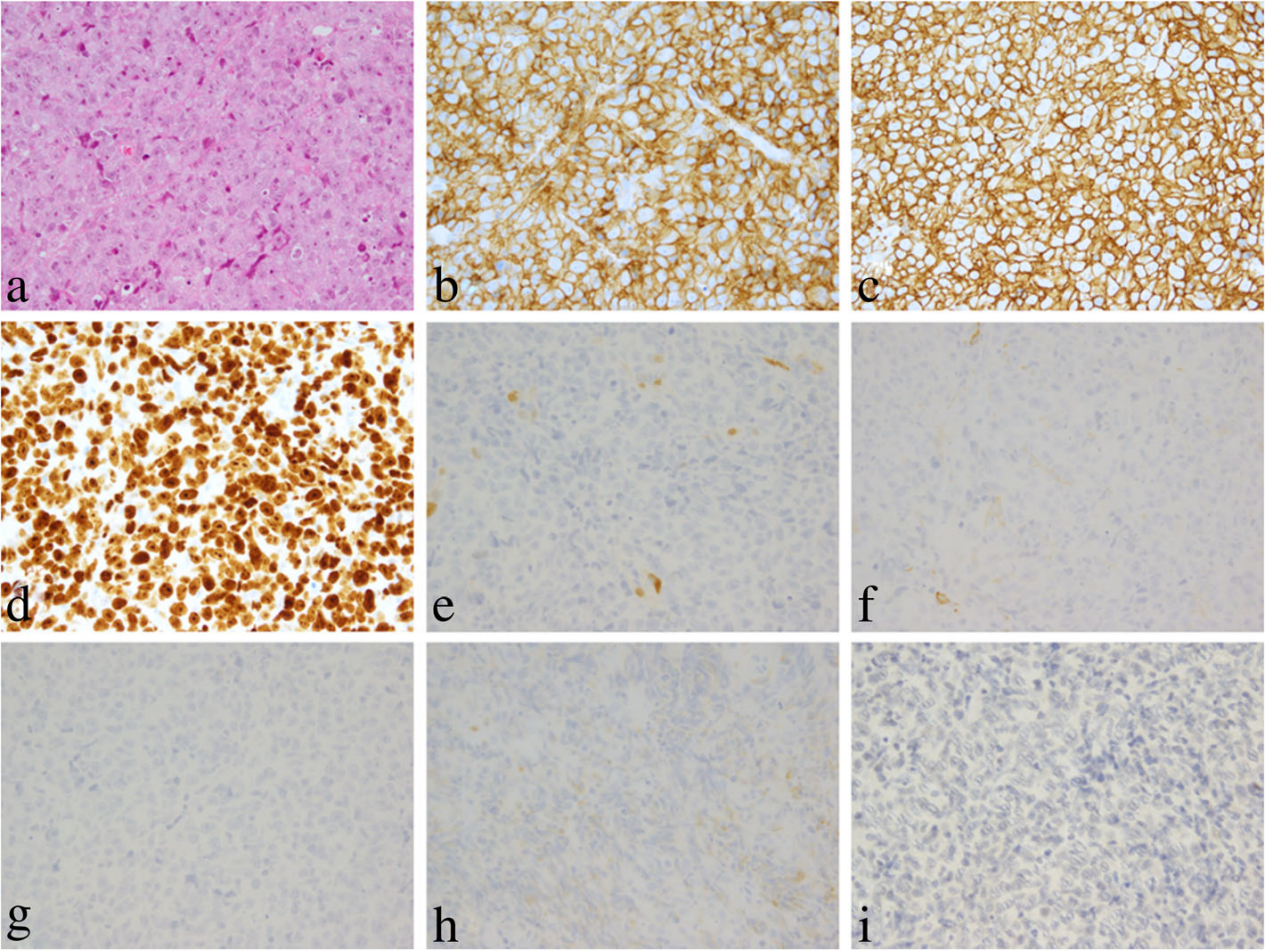
**a**, Histology of biopsy specimens from ulcerated stomach lesions showing infiltration of large atypical lymphocytes (H&E × 400). **b**, Immunohistochemical studies revealing the expression of CD5 (× 400), **c,** CD20 (× 400), **d**, and Ki-67 antigen (× 400), but the absence of **e**, cyclin D1 (× 400), **f**, CD10 (× 400), **g**, CD30 (× 400), **h**, BCL-2 (× 400), and **i**, the EBV (using EBER-ISH) (× 400)

**Fig. 3:**
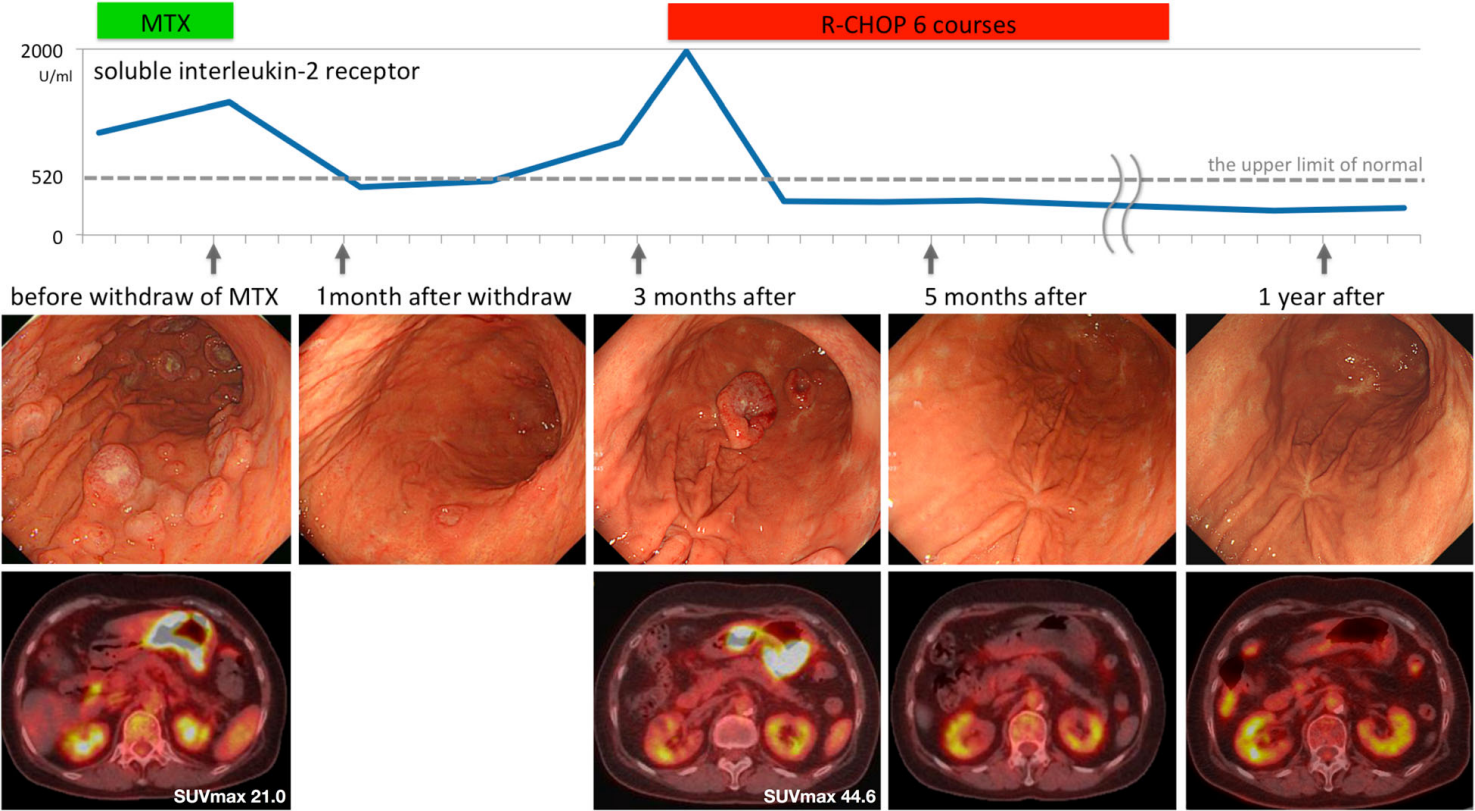
Clinical causes: We observed changes in MTX-LPD symptoms in the stomach and duodenum with regard to the levels of sIL-2R, and EGD and PET-CT findings

EGD performed at the time of admission to our hospital revealed multiple “dish-like” lesions in the stomach and duodenum (Fig. 1a, d). Indigo carmine spraying revealed that the lesion elevation was relatively steep, the surface structure was equivalent to that of the background mucosa, and ulceration with white coat was observed in the central part of the lesion (Fig. 1b). Narrow band imaging revealed meandering irregular microvessels without loops (Fig. 1c). These results suggest that a solid tumor growing from the submucosa was ulcerated and exposed at the central part of the lesion. The histology of biopsy specimens obtained from the ulcerated lesions showed infiltration of large atypical lymphocytes. Immunohistochemical studies revealed the expression of cluster of differentiation (CD)5, CD20, and Ki-67 antigen, but the absence of cyclin D1, CD10, CD30, B-cell lymphoma (BCL)-2; Epstein–Barr virus (EBV)-encoded small RNA in situ hybridization (ISH) demonstrated that the EBV was absent (Fig. 2a–i). We carried out positron emission tomography–computed tomography (PET–CT) to evaluate the extent of disease. PET–CT showed abnormal uptake of radioactive tracers in the stomach, duodenum, and a few adjacent nodes, with a maximum standardized uptake value of 21.0 (Fig. 3). Based on these findings, and along with the patient’s history of RA treated with MTX, she was diagnosed with MTX-LPD showing features of stage II_1_ diffuse large B-cell lymphoma (DLBCL) (Lugano classification).

Initial management consisted of the discontinuation of MTX, which resulted in symptom improvement and reduction of sIL-2R level. Two weeks after the withdrawal of MTX, the lymphocyte count increased from 2375/μl to 5616/μl (52%). EGD conducted 1 month after discontinuation revealed a reduction in the number of lesions with some scarring (Fig. 3). Pathological findings confirmed residual tumor cells. Three months after discontinuation, epigastric distress worsened and the sIL-2R level reached 1973 IU/mL. A third EGD showed the recurrence of multiple lesions. PET–CT showed abnormal uptake of radioactive tracers with a maximum standardized uptake value of 44.6 in the stomach (Fig. 3). We suspected MTX-LPD relapse and started six courses of rituximab, cyclophosphamide, hydroxydaunorubicin, oncovin, and prednisolone (R-CHOP) chemotherapy. After starting chemotherapy, her symptoms and the sIL-2R level improved rapidly. We carried out EGD and PET–CT 1 month from chemotherapy commencement that revealed the disappearance of the lesions and no evidence of lymphoma on pathological evaluation. One year after the cessation of chemotherapy, she remained asymptomatic, and the complete response of MTX-LPD was confirmed on the EGD, pathological examination, and PET–CT (Fig. 3).

## Discussion and conclusions

We described the case of a 70-year-old woman with MTX-LPD of the stomach and duodenum. In this case, disease regression was temporarily achieved after the cessation of MTX treatment, but it subsequently recurred and complete response was only achieved after chemotherapy.

RA patients have a twofold to fourfold increased risk of developing lymphoma compared to that of the general population [5]. MTX, the “anchor drug” for RA, is considered to be a major cause of lymphoproliferative disorders. The pathogenesis of MTX-LPD is incompletely understood, but studies have suggested that the “hyper-immune” state of RA and the immunosuppressive state associated with MTX might contribute to MTX-LPD development. For patients taking MTX, a shorter interval between the diagnosis of RA and LPD in MTX-LPD than in non-MTX-LPD (median, 132 vs. 240 months, respectively) has been documented [6], and the withdrawal of MTX treatment results in the spontaneous remission of LPD in 25–60% of patients taking MTX [2, 4, 6]. In an observational study about 102 cases of MTX-LPD, 47 patients were only withdrawn MTX without any additional treatment of LPD. In 28 of 47 (60%) patients, spontaneous remission occurred and continued for 3–84 months (median, 17). 13 of 28 (46%) patients required chemotherapy because of recurrence or residual disease [7].

A large observational cohort study of 18,572 patients with RA [8] suggested that MTX contributed to a small increased risk of LPD development, with a standardized incidence ratio (SIR) of 1.7 (95% confidence interval (CI), 0.9–3.2). In a nested case–control study in Japan involving 5753 RA patients (28 patients in the MTX-LPD group and 125 patients in the MTX non-LPD group), Kameda et al. [9] reported that the mean dose of MTX was higher in the MTX-LPD group (8.4 [range, 5.9–10.0] mg/week) than in the non-LPD group (7.0 [5.0–8.6] mg/week). They suggested that a higher mean MTX dose is an independent risk factor for LPD development in RA patients. Studies have suggested that immunosuppressants other than MTX and biological preparations (e.g., infliximab, etanercept) can also induce LPD. In a 5-year cohort study of 1252 patients with severe psoriasis patients treated with cyclosporine, Paul et al. reported [10] that the incidence of leukemia was significantly elevated in the cohort compared to that in the general population (SIR; 7.3, 95% CI: 1.5–21.5). Hasserjian et al. reported 18 cases of lymphoproliferative disorders caused by biological preparations (e.g., Infliximab, Adalimumab, Etanercept) for autoimmune diseases (e.g., RA, psoriasis, inflammatory bowel disease) [11]. Only 6 patients had received prior treatment with MTX among them.

EBV positivity might also contribute to MTX-LPD development. EBV has been implicated in Hodgkin’s lymphoma, Burkitt’s lymphoma, gastric carcinoma, and nasopharyngeal carcinoma [12]. Studies have shown that the monoclonal proliferation of host cells by EBV involves 3 steps: expression of the oncogenes of EBV, genetic/epigenetic changes in the host, and dysfunction of the immune system [13]. Hoshida and co-workers [6] found that the prevalence of EBV in RA patients with LPD was significantly higher than that in sporadic LPD (27.6% vs. 9.9%).

Almost 50% of MTX-LPD cases show remission merely by the withdrawal of MTX treatment [4]; thus, the first-choice treatment of MTX-LPD is the rapid cessation of MTX. However, subsequent recurrence of MTX-LPD has been reported in 18–45% of patients, and chemotherapy is indicated in cases of recurrence or in those not reaching remission after > 3 months [7, 14, 15]. Recent studies suggest that the pattern of lymphocyte count transition during withdrawal of MTX is associated with the spontaneous regression of MTX-LPD. Saito et al. reported that the lymphocyte count increased more than 220/μl in the regressive group compared to less than 150/μl in the persistent group at 2 weeks after the withdrawal of MTX [16]. Inui et al. reported the lymphocyte count increased 600/μl on an average at 2 weeks after the withdrawal of MTX in 20 cases of MTX-LPD [17]. Even our case was consistent with the findings that the lymphocyte count increased from 2375/μl to 5616/μl in 2 weeks, and that the spontaneous remission of MTX-LPD was observed.

Other studies have shown a high prevalence of spontaneous remission to be associated with EBV-positivity and a non-DLBCL histology type. Older age (> 70 years) and a DLBCL histology type are predictive factors of shorter survival [7]. In MTX-LPD patients with a DLBCL histology type, five-year survival is 74%, and five-year progression-free survival is 65% [14]. CD5-positive DLBCL is closely associated with aggressive clinical features and parameters; thus, the overall International Prognostic Index score of CD5-positive DLBCL is significantly higher than that of CD5-negative DLBCL [18].

Few reports have focused on the endoscopic features of gastric or duodenal lesions in MTX-LPD. Ikeda et al. [19] reported MTX-LPD of the stomach as DLBCL, which featured multiple elevated lesions with dish-like ulcers in the lower body of the stomach. Satoh et al. [20] reported MTX-LPD as DLBCL, presenting as a single ulcerative lesion resembling a Borrmann type-II advanced gastric cancer. The endoscopic pattern of gastric DLBCL varies; in most cases, a single or multiple dish-like ulcerative lesions at the gastric body or fundus are noted [21]. Characteristic features of MTX-LPD include prompt disappearance of lesions after discontinuing MTX, and achievement of spontaneous remission.

Our case was classified as carrying a low rate of spontaneous remission and aggressive characteristics because of the patient’s advanced age, DLBCL histology type, CD5-positivity and EBV-negativity. In only a month after cessation of MTX treatment, her symptoms improved, and EGD showed that most lesions had disappeared. Subsequently, however, the symptoms exacerbated and the sIL-2R level increased. Repeat EGD revealed regrowth of lesions, and she was started on R-CHOP chemotherapy, which resulted in complete response.

Various new drugs with high efficacy, such as biological preparations, have been developed for treating RA. However, these drugs are very expensive, and it is highly likely that MTX—which is inexpensive and highly effective—will remain the first-line drug. Hence, future studies must examine the mechanism underlying the development of MTX-LPD, and the elucidation of this mechanism will help in preventing the occurrence of MTX-LPD.

In lymphoma patients treated with MTX, the first-choice therapy is the withdrawal of MTX treatment if MTX-LPD is suspected. Even after the remission of MTX-LPD, careful observation is important, and if the disease recurs, chemotherapy should be commenced promptly.

## Abbreviations

BCL: B-cell lymphoma
CD: cluster of differentiation
CI: confidence interval
DLBCL: diffuse large B-cell lymphoma
EBV: Epstein–Barr virus
EGD: esophagogastroduodenoscopy
ISH: in situ hybridization
MTX-LPD: Methotrexate-associated lymphoproliferative disorder
PET-CT: positron emission tomography–computed tomography
RA: rheumatoid arthritis
R-CHOP: rituximab, cyclophosphamide, hydroxydaunorubicin, oncovin, and prednisolone
sIL-2R: soluble interleukin-2 receptor
SIR: standardized incidence ratio
